# The Microfoundations of Organizational Risk

**DOI:** 10.1111/risa.70092

**Published:** 2025-07-26

**Authors:** Emma Soane

**Affiliations:** ^1^ Department of Management London School of Economics and Political Science London UK

**Keywords:** culture, microfoundations, organizational risk, personality, risk‐taking

## Abstract

Organizational risk is the possibility of events preventing the achievement of objectives and disrupting organizational viability. Developing understanding of organizational risk is necessary to allow realization of opportunities and protection from harm. However, much existing theorizing focuses on either a higher level of analysis, for example, studies of organizational risk culture, or a lower level of analysis, such as studies of individual perception, personality, and risk‐taking. One way to advance theorizing involves connecting both levels of analysis. These connections are central to a microfoundations perspective that suggests organizational phenomena can be understood by linking macrolevel contexts with microlevel contexts and actions. I draw on this perspective to develop a model of organizational risk and explain how cross‐level processes connect macro‐ and microlevel concepts. I focus on the organizational psychology literature that encompasses higher and lower levels of analysis to select and examine relevant concepts. I explain how organizational cultures create contexts for individual risk‐taking that are homogeneous when constraints are strong and directional or variable when constraints are weak and ambiguous. These behaviors aggregate within and across units to influence organizational risk. Individual risk‐taking also influences organizational risk via autonomy and discretion. In developing the model, I show how theories of cross‐level processes extend understanding of organizational risk. I discuss implications for advancing theorizing about organizational risk by encompassing its microfoundations and linking them with managerial actions and objectives. Future research could examine these mechanisms through empirical studies and shed light on how leaders influence processes and change organizational risk.

## 1 Introduction

Organizational risk concerns possibilities for negative outcomes at the level of the organization that disrupt their viability (Bromiley et al. 2001). Prior research suggests organizational risk has internal sources (Pettersen Gould 2021; Taarup‐Esbensen 2019; Turner and Pidgeon 1997). Understanding organizational risk is thus critical to modeling accidents and disasters (Reason 1997, 2016) as well as outcomes such as growth (Hoskisson et al. 2017), performance (Hardy et al. 2020), reputation (Bryce et al. 2024), and ethics (Bouchard and Dion‐Labrie 2024).

One line of enquiry examines how the norms and practices embedded in organizational culture influence organizational risk (Bockius and Gatzert 2024). For example, there is widespread agreement that the cultural norms and practices shaping the relationship between speed and safety are key to understanding such influence. Emphasizing speed creates organizational risk, whereas emphasizing safety reduces it (Leaver and Reader 2019). A second line of enquiry considers the role organizational actors play in making and enacting decisions that have consequences for organizational risk (Gephart et al. 2009). Individual differences in perceptions, personality, and propensity influence orientations to risk and risk‐taking (Lauriola and Weller 2018). Furthermore, individual‐level risk‐taking has far‐reaching implications for organizational risk when enacted with discretion (Li and Tang 2010).

Theories have emerged at different levels of analysis and use different mechanisms to explain organizational risk. While studies of individual‐ and organizational‐level determinants of organizational risk are advancing (Bockius and Gatzert 2024; Pan et al. 2017), integrating theorizing is a challenge (Hitt et al. 2007). Theorizing at a specified level of analysis creates insights unique to that level (Kozlowski and Klein 2000). For example, a wealth of evidence supports connections between individual differences and risk‐taking behavior (Joseph and Zhang 2021). However, precise explanations of the drivers of behavior and the impact of behavior on organizational risk require additional theorizing, with particular attention to the cross‐level processes that connect levels of analysis (Cowen et al. 2022). Moreover, organizational processes and individual behaviors may increase as well as decrease risks, creating additional challenges for modeling organizational risk (Pettersen Gould 2021). Thus, I pose two questions: What are the microfoundations of organizational risk? What are the cross‐level mechanisms that connect micro‐ and macrolevel phenomena to explain organizational risk?

To address these questions, I draw on the microfoundations perspective that proposes organizational contexts create conditions for actions that aggregate to influence organizational phenomena, the corollary being organizational phenomena are best understood by looking at their lower‐level antecedents (Barney and Felin 2013; Felin et al. 2015; Felin and Hesterly 2007). Central to the microfoundations perspective is the notion that macro‐ and microlevel phenomena are connected by a sequence of situational, action formation, and transformational mechanisms, respectively (Cowen et al. 2022). To build on this framework, I use theories from psychology that encompass organizational‐ and individual‐level concepts as well the connections between them. I examine organizations using the concepts of culture (Schein 2017) and risk (Hoskisson et al. 2017), and individuals through the lenses of perceptions, personality, propensity, and risk‐taking behavior (Highhouse et al. 2022; Joseph and Zhang 2021; Mata et al. 2018; Nicholson et al. 2005). Each of these concepts is the subject of abundant research and clear theorizing (Tett and Fisher 2021; Tett et al. 2021).

With this study, I make contributions to the literature concerned with the microfoundations movement (Barney and Felin 2013; Felin et al. 2015). I draw on established concepts and focus on developing theorizing about the connections between them that are necessary to advancing microfoundations theorizing (Cowen et al. 2022). Additionally, I contribute to the risk science literature (Aven, 2024b; Aven and Zio 2021) by showing how theories from psychology constitute part of the interdisciplinary theorizing required to understand the complexities of organizational risk.

## 2 Literature Review

### 2.1 The Microfoundations Perspective

**FIGURE 1:**
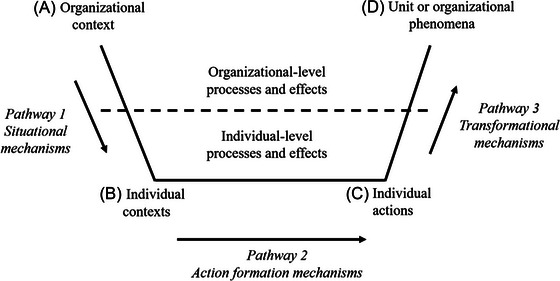
The microfoundations model.

Coleman (1990) argues macrolevel explanations of macro phenomena are insufficient because the micro phenomena that underpin them are not theorized. The microfoundations perspective addresses this problem by layering micro and macro phenomena, with individuals at the base of the model (Little 1991). Microfoundations studies play a key role in addressing complex challenges by expanding theorizing and offering novel insights into the multilevel processes that influence organizational outcomes (Cowen et al. 2022). By taking a multilevel approach, the microfoundations perspective recognizes individual differences in motives and goal attainment contribute to organizational heterogeneity and enhanced performance through value creation (Eisenhardt et al. 2010; Foss and Lindenberg 2013; Lindenberg and Foss 2011). Figure 1 depicts the core elements of the microfoundations model (Coleman 1990).

As a consequence of its theoretical and practical utility, research taking a microfoundations perspective is burgeoning, encompassing a wide range of topics and discourses (Felin et al. 2015). These studies advance explanations of organization‐level phenomena including cybersecurity (Kostyuk and Wayne 2021), audit (Power 2021), design thinking (Magistretti et al. 2021), technology and innovation (Palmié et al. 2023), and knowledge sharing (Foss and Pedersen 2019).

There are implications for organizational risk. For example, Power's (2021) examination of the audit society considers how the production of performance accounts via audit trails connects individuals and organizations via cross‐level processes. Audit and risk management are fundamentally interconnected (Power 2004), thus modeling its microfoundations highlights the interplay between organizational risks, such as the push and pull between internal and external audit pressures, and individual risks, such as employment risk arising from challenges in adapting to new procedures or the legal and financial risks arising from disclosures. These processes influence organizational risk at it pertains to supported or diminished values that impact on capacity to reach goals.

I build on microfoundations studies by exploring key concepts and cross‐level processes that influence organizational risk. In line with prior research (Hitt et al. 2007), I consider risk as a phenomenon that has meaning at the individual and organizational levels of analysis. For example, the potential for financial and reputational losses to realize after a merger or acquisition are organizational risks that are distinct from the individual risks for employees who may experience strain and job insecurity (Degbey et al. 2021; Hughes et al. 2020). I begin by exploring how organizational cultures create contexts for individual actions.

### 2.2 Organizational Culture and Norms

Organizational contexts are the starting point for a microfoundations perspective on organizational risk. A key element of context is organizational culture that is defined as:

The accumulated shared learning of that group as it solves its problems of external adaptation and internal integration … This accumulated learning is a pattern or system of beliefs, values, and behavioral norms that come to be taken for granted as basic assumptions and eventually drop out of awareness. (Schein 2017, 6)

A cultural lens offers subtle and deep insights into the norms that reflect organizational systems and influence behavior (Guldenmund 2010). Prior research within the psychology domain shows two aspects of culture that shape individual‐level risk‐taking and have implications for organizational risk.

One aspect is the character of culture in relation to risk (Palermo et al. 2017) and safety (Guldenmund 2000; Pidgeon 1998) given that accidents are an indication that organizational processes have enabled risk to materialize as harm (Pate‐Cornell and Murphy 1996). Organizational cultures may also support risk‐taking in the pursuit of innovation (Frishammar et al. 2019). Such cultures comprise processes, values, behaviors, and recognition that foster the generation and implementation of new ideas (Rao and Weintraub 2013) and learning (Vey et al. 2017). By contrast, cultures characterized by a fixation on current paradigms and practices constrain innovation and reduce risk‐taking (Stempfle 2012).

A cultural focus on risk also creates challenges. The financial crisis of 2008 and its ensuing effects have drawn attention to the culture evident in financial services organizations and how excessive rewards and risk‐taking have led to damaging consequences for customers as well as wider society (Butler and Brooks 2024). Norms that favor production speed rather than safety increase the likelihood of accidents (Leaver and Reader 2019). A review of high‐risk sectors suggests some organizational cultures normalize practices that contribute to disasters, such as insufficient training or ineffective management, and hamper efforts to deal with failures, for example, by normalizing inaction (Hald et al. 2021).

Culture also promotes safety by upholding prescriptive norms concerning valued behaviors, such as safe working practices, that reduce possibilities for risk to materialize as harm (Dannals and Miller 2017). Likewise, culture supports efforts to achieve safe environments with low accident rates despite the hazards involved, as seen in the aviation sector where norms emphasize adherence to protocols and attention to safety (Kirwan et al. 2018; Noort et al. 2019).

The creation of subcultures is a second aspect of culture that influences contexts and individual behaviors in ways that are key to microfoundations theorizing about organizational risk. Although cultural unity is possible in small organizations or organizations strongly bound by common goals (Collins and Porras 1994; Mintzberg 1979), subcultures reflecting the differing interests of groups of organizational members are common (Schein 1996). Large, diverse, or multi‐divisional organizations typically comprise sets of interacting subcultures due to their complexity (Reason 1998). For example, a study of the nuclear industry in Spain, an archetypal example of a high‐risk sector, shows clusters of subcultures, including a constructive culture involving cooperation, teamwork, and service quality, a passive/defensive culture that pressures employees to please those in authority, and an aggressive/defensive culture that fosters opposition and competition. Moreover, patterns of subcultures differ across locations and organization types (e.g., nuclear power plants and nuclear public companies) (Badia et al. 2020).

In‐depth examination of subcultures shows two distinct forms. Cultural differentiation concerns inconsistent interpretations of themes, practices, and forms, suspicion of claims of cultural consensus, and creates a view that is clear within subcultures and ambiguous across subcultures. Cultural fragmentation describes the multiplicity of perspectives that emerge in complex contexts and do not coalesce into a coherent whole (Martin 1992). Indeed, studies of differentiation and fragmentation shed new light on the impact of culture on organizational risk. For example, an examination of cultural fragmentation in a study of leadership and employee engagement reveals pockets of psychosocial risks, including bullying and counter‐productive behaviors, that a focus on cultural integration could overlook (Latta 2019). Both differentiation and fragmentation are sources of organizational risk when they loosen connections between behaviors and procedures, one outcome being practical drift whereby processes and actions gradually deviate from procedures (Snook 2000). Thus, cultures and subcultures create specific types of contexts for individual actions.

Normative influences connect organizations with individuals by creating norms, processes, and practices that orient behavior in particular directions, including risk‐taking. This form of influence is explained by situational strength theory (Mischel 1977) that considers how combinations of organizational and relational structures produce regularity or variability (Meyer and Dalal 2009). Strong situations are characterized by constraints that define expectations, cues that signal the importance of conformity, clarity of goals, and consequences that are positive when behavior is aligned with expectations and negative when behavior diverges from expectations. Such situations generate powerful norms, goals, rewards, and sanctions that limit expressions of individual preferences (Meyer et al. 2010). By contrast, weak situations are characterized by decentralized structures, localized practices, discretion over decision‐making, and ambiguity (Barrick and Mount 1991). Weak situations make individual‐level responses salient, rendering situations open to perception and interpretation, and create opportunities for the expression of personality (McCrae and Costa 1999).

The corollary is that cultures characterized by strong situations create behavioral homogeneity. Whether behavior focuses on reducing or increasing organizational risk depends on specific cultural norms and practices. Alternatively, cultures characterized by weak situations create behavioral variability and opportunities to enact individual differences. Thus, culture is a key aspect of organizational context (Concept A in Figure 1). Normative influences represent Pathway 1 in Figure 1 that connects organizational contexts with individual contexts for action.

### 2.3 Individual Differences and Risk‐Taking

The second aspect of microfoundations theorizing concerns individual‐level concepts (Concept B in Figure 1), including perception, personality, and propensity that influence individual risk‐taking (Pathway 2 and Concept C in Figure 1, respectively). Risk perception, “a person's subjective judgement or appraisal of risk” (Society for Risk Analysis 2018, 8), is key to explaining how influence occurs. Scholars agree that appraisals of risk comprise perceptions of loss and gain that typically differ relative to each other (Slovic et al. 2016). A key insight is that risk‐taking is more likely when perceived benefits exceed perceived losses (Siegrist and Árvai 2020).

Demographic characteristics, particularly age and sex, are one source of influence on risk perceptions. Evidence from 11 countries suggests risk‐taking peaks in late adolescence and then reduces with maturity (Duell and Steinberg 2019; Duell et al. 2018). A meta‐analytic study shows a general pattern of greater risk‐taking among men than women, with divergence being greater in some areas, notably intellectual and physical risk‐taking, than others, such as smoking (Byrnes et al. 1999). These effects occur via perceived costs and benefits (Frey et al. 2021) that are processed in different regions of the brain (Levin et al. 2012).

Perceptions are also influenced by personality, “the enduring characteristics and behavior that comprise a person's unique adjustment to life, including major traits, interests, drives, values, self‐concept, abilities, and emotional patterns” (American Psychological Association n.d.). Studies show clear relationships between personality, perceptions, and risk‐taking, with sensation‐seeking being a well‐studied antecedent (Lauriola and Weller 2018). Sensation‐seeking is “the seeking of varied, novel, complex and intense sensations and experiences that motivates the willingness to take physical, social, legal, and financial risks for the sake of such experience” (Zuckerman 1994, 27). Empirical studies support this assertion, showing sensation seeking is associated with risk‐taking in a range of domains, such as health and financial risk‐taking, (Nicholson et al. 2005) as well as specific behaviors, such as high volumes of trading in financial markets (Grinblatt and Keloharju 2009). Although such risk‐taking brings thrills and excitement, possibilities for harm are managed through planning and learning, for example, scuba‐diving training or preparing for a performance (Woodman et al. 2013).

Impulsivity provides a different mechanism that explains risk‐taking in terms of perceived benefits outweighing perceived costs due to low levels of conscientiousness (preferences for spontaneity), low agreeableness (tendencies to be tough‐minded), and low self‐control (Joseph and Zhang 2021). The negative emotions associated with trait neuroticism (anxiety and worry) also alter perceptions of losses and gains, promoting risk‐taking when the latter exceeds the former (Soane et al. 2010) and inhibiting risk‐taking when neuroticism (the tendency to be anxious) and negative emotions enhance perceived dangers (Weller and Tikir 2011).

Moreover, individual differences exist in risk propensity, which is the tendency to focus more on the potential positive outcomes of actions with uncertain consequences than on the potential negative outcomes (Highhouse et al. 2022). Risk propensity has a general effect on risk‐taking (Zhang et al. 2019) and specific influences risk‐taking in particular domains, such as health and finance (Soane and Chmiel 2005). These effects are over and above those of personality traits (Highhouse et al. 2022).

The relevance of individual differences to a microfoundations approach to organizational risk lies in their interactions with contexts that influence the expression of traits in behavior. Such interactions are explained by trait activation theory (Tett and Burnett 2003; Tett and Guterman 2000) that proposes traits are activated by demands relating to organizational cues (e.g., a norm of respect for hierarchy), social cues (e.g., a request to collaborate with team members), and task cues (e.g., a task that requires independent work) (Tett and Burnett 2003).

Although the core trait activation theorizing considers risk‐taking (Tett and Guterman 2000), subsequent studies shift the focus to concepts such as performance and motivation. Evidence shows connections between personality and behavior are strengthened when traits are activated, and expression of individual traits is organizationally valuable. For example, managers’ achievement‐striving traits are activated by goal difficulty and expressed in terms of commitment to goal attainment that contributes to achieving organizational objectives (Aksoy and Bayazit 2022). Conversely, personality‐behavior associations are weakened when traits are suppressed by cues that demand specific behaviors that have organizational rather than individual value (Tett and Fisher 2021; Tett et al. 2013). A consequence is that managers shape trait expression by generating cues that influence how traits are enacted in behaviors and emphasizing the value of behaviors that increase possibilities for attaining organizational objectives (Tett et al. 2021).

The same principles apply to sensation seeking and other risk‐relevant traits, their activation by situations, and subsequent expression in risk‐taking. Moreover, activation processes also apply to individual characteristics that are not traits, including propensities (Tett et al. 2013). The specific nature of activation processes creates variability in behavior within and across individuals (Judge and Zapata 2015). Thus, the individual behaviors represented by point C in Figure 1 are homogeneous when shaped by cultures creating strong situations and variable when shaped by cultures creating weak situations that are perceived in accordance with individual traits and risk propensity, then activated and expressed in differing levels of risk‐taking.

### 2.4 Effects of Individual Risk‐Taking on Organizational Risks

Elucidating the cross‐level mechanisms that explain how individual‐level attributes influence organizational‐level outcomes (Pathway 3 and Concept D in Figure 1) is key to microfoundations theorizing (Barney and Felin 2013; Felin et al. 2015). Cross‐level effects are understood in terms of composition processes concerning the linear aggregation of variables, whereby lower‐ and higher‐level constructs are equivalent, and compilation processes concerning the nonlinear aggregation of variables, whereby the higher‐level construct is not equivalent to its lower‐level counterpart (Chan 1998; Kozlowski and Klein 2000). For example, culture is an organizational construct that is distinct from the people within it (Schein 1996), whereas personality is an individual‐level construct that cannot describe organizations (LePine et al. 2011). Similarly, organizational risk is a higher‐level phenomenon that is influenced by individual risk‐taking yet not the same as it. Thus, I focus on compilation processes and identify three mechanisms that explain the cross‐level relationships between individual‐level risk‐taking and organizational risk.

The first mechanism concerns the homogeneity within and across organizational units that arises from internal and external sources of pressure. Individuals are more comfortable with others when they share beliefs, values, and characteristics (Lazarsfeld and Merton 1954), and will make adjustments to create sharedness (Heider 1958). Social‐tuning mechanisms enable people to develop strong social bonds through the perception and expression of similarity (Shteynberg 2010). These processes influence organizations by shaping the attraction and selection of people whose characteristics and goals are coherent with the organization as well as the attrition of people who are not compatible (Schneider 1987), thus increasing organizational homogeneity over time (Schneider et al. 1995). Similar pressures also create strong situations that constrain the expression of individual differences (Mischel 1977). Doing so creates possibilities for aligning risk‐taking with organizational objectives.

For example, homogeneous and high levels of risk‐taking are apparent within small organizations characterized by venturesome objectives in hazardous environments, such as the deep‐sea exploration company, OceanGate. Its CEO, Stockton Rush, who claimed to innovate on safety, led excursions on the submersible, Titan, that tragically imploded due to structural weaknesses while diving to the Titanic wreck (Bogel‐Burroughs et al. 2023). Homogeneous and low levels of risk‐taking are also evident. For example, in organizations that manufacture medical diagnostic tools and devices where safety is their core focus. This approach is exemplified in Abbott, the first organization licensed to test blood for the HIV virus and a key player in producing effective tests for the COVID‐19 virus (Perchetti et al. 2021).

More generally, a strong and coherent focus on risk management and safety is typified by high reliability organizations that attempt to build almost error‐free systems (Le Coze et al. 2014). Such organizations create strong cognitive and social demands to foster high levels of attention to risk as well as safety, cross‐organizational networks, and a focus on learning (Bigley and Roberts 2001; Roberts et al. 1994). These organizations are adaptable, showing responsiveness to situational features as well as regulatory and societal demands (La Porte 1996). Together, these features create collective mindfulness that perpetuates high reliability (Christianson et al. 2011; Weick et al. 1999). Studies in this domain have produced a distinct line of enquiry as well as developing an understanding of organizations as connected systems of individual‐level processes and behaviors that enable consistently low levels of organizational risk, high levels of safety and performance through responsiveness and coherence (Le Coze 2019b). Thus, individual risk‐taking and organizational risk are connected by strong directional approaches to risk embedded in norms, processes, and practices.

A second cross‐level mechanism that connects individuals with organizations centers on the effects of variability in individual‐level risk‐taking caused by subcultures, weak situations, individual differences, and activation effects. Variability arises from pressures that support differences within and across organizational units (Ostroff and Fulmer 2014). Optimal distinctiveness theory (Brewer 1991, 2007) explains within‐unit variability by proposing two concurrent processes operate within groups. A process focusing on between‐person similarities to create social bonds and reduce isolation is complemented by a process focusing on between‐person differentiation that creates exclusivity. Organizational pressures also create variability across units due to needs for specialization and functional divisions that enable organizations to be situationally responsive and innovative (Burns and Stalker 1961). Such processes foster differences in unit‐level characteristics and performance (Stewart 2003).

Between‐person and between‐unit variability is interpreted in terms of patterns (Ostroff and Fulmer 2014). A linear pattern describes variability in terms of extent, from low to high. Low variability is akin to the notion of organizational homogeneity discussed above, whereas high variability indicates a range of risk‐taking behaviors that differ in their possible consequences. Variability in risk‐taking across organizations creates opportunities for achievements as well as increasing exposure to losses (Power 2004). For example, traders in investment banks are hired to take risks yet their risk‐taking varies within and across teams. Managers assume a combination of preferences and incentives creates sufficient risks and rewards to meet organizational objectives (Fenton‐O'Creevy et al. 2005).

However, further insights into variability are necessary if harm is to be reduced and opportunities are to be realized effectively (Elton et al. 2010). Such insights can be achieved by considering how variability creates patterned profiles that are meaningful at a higher level (Nunnally 1995). Profiles describe the differential characteristics of organizational units in ways that create configurations (Doty et al. 1993) and explain their functioning (Ostroff and Fulmer 2014). For example, a pattern of organizational climate characterized by systems that support employees has a positive association with financial outcomes, whereas a strategic climate focused on internal management processes does not (Schulte et al. 2009).

Thus, differentiation between units can be understood in terms of patterned variability and configurations that have implications for organizational risk. Multidivisional organizations comprise divisions with particular functions (Hoskisson 1987). Often, these functions have different orientations to risk. For example, divisions focused on research and development or operations have different constructions of the meaning of risk and how it affects their functioning (McKenna 2001). Such configurations play a role in explaining cross‐level processes by showing how pressures for homogeneity within units and variability across units create profiles that influence organizational risk.

A third form of cross‐level process occurs when key individuals exercise authority and discretion. Upper echelons theory (Hambrick 2007) suggests CEOs influence their organizations by perceiving situations in characteristic ways and enacting strategic choices including risk‐related issues, such as innovation and diversification. These choices affect organizational performance, profitability, growth, and survival (Hambrick et al. 2015). CEOs’ influence is strong when they have high levels of autonomy and when organizational structures are weak, enabling CEOs to express their preferences (Neely et al. 2020). For example, CEO hubris, a combination of traits and beliefs that generate excessive optimism and self‐confidence (Hiller and Hambrick 2005), is positively associated with organizational risk‐taking especially when CEOs have discretion and when they are also chairs of their organization's boards (Li and Tang 2010).

Moreover, CEO positive self‐concept is positively related to strategic risk‐taking as well as other risky activities, including acquisitions and product innovation (Wang et al. 2016). CEOs with higher trait extraversion and openness bear higher levels of equity risk (i.e., the equity wealth component of the firm‐specific wealth at risk of loss in the event of failed risk‐taking; Wiseman and Gomez‐Mejia 1998) and engage in more strategic risk‐taking (Benischke et al. 2019). Conversely, CEOs who experience negative emotions avoid organizational risks (Delgado‐García et al. 2010; Mannor et al. 2016).

Moreover, individual‐level risk‐taking occurs throughout organizations, with each act creating possibilities for risk to materialize as organizational harm. In some cases, risk created by one person is sufficiently extreme to shake an entire organization, as was evident when Nick Leeson's unauthorized trading brought down Barings Bank and in other “rogue trader” events (Greener 2006). Although none of these traders was a senior executive, their autonomy played a key role in enabling substantial risk‐taking (Wexler 2010). Additionally, risk‐taking behavior that is less consequential in isolation may aggregate and become influential at the organizational level. For example, both a surgeon who makes a surgical error and an administrator who makes errors in appointment bookings harm their patients and their hospital's reputation (Moffatt‐Bruce et al. 2016). The accumulation of such actions has an aggregate effect whereby more risk arising from individual‐level actions creates more risk at the organizational level (Baird and Thomas 1985; DuHadway et al. 2018).

The corollary is that organizational risk is an outcome of compilation processes that explain how individual‐level risk‐taking aggregates to create homogeneity and variability within and across units. Organizational risk is aligned with individual‐level risk‐taking in strength and direction when pressure for homogeneity is strong, as seen in cohesive cultures that orient people and organizations in relation to risk (Hald et al. 2021; Reader et al. 2015), strong situations (Judge and Zapata 2015), and powerful unit‐level processes that foster shared objectives among individuals (Doty et al. 1993; Ostroff and Fulmer 2014). Organizational risk also arises from individual actions that are variable within and across units in ways that form coherent profiles (Schulte et al. 2009). Moreover, organizational risk is shaped by senior executives and influential actors whose risk behavior and discretion confer organizational‐level impact (Hoskisson et al. 2017; Li and Tang 2010). Table 1 summarizes these key concepts and the connecting processes that explain organizational risk.

**TABLE 1:** Summary of cross‐level processes and mechanisms.

Cross‐level process	Mechanism
Connecting organizational culture with individual‐level contexts for action: normative influences	Normative influences uphold core values, for example, ethics (Kaptein 2009), safety (Hald et al. 2021; Le Coze 2019a), and innovation and adaptability (Andersson et al. 2019; Deverell and Olsson 2010). Situations may be strong, creating behavioral homogeneity, or weak, creating behavioral variability (Mischel 1977).
Connecting individual‐level contexts with individual‐level behaviors: activation processes and expressions of perceptions, traits, and propensity	Individuals respond to task, social, and organizational cues that activate the expression of traits and other individual differences in risk‐taking (Tett and Burnett 2003). Weak situations permit enactment of individual differences in risk‐taking (Frey et al. 2021; Mata et al. 2018).
Connecting individual‐level actions with organizational risk: aggregation and impact	Homogenous individual‐level risk‐taking aggregates within and across units to influence organizational risk (Le Coze et al. 2014). Variable individual‐level risk‐taking aggregates to influence organizational risk in ways that may be patterned across units or highly variable within and across units (Ostroff and Fulmer 2014). Individuals with authority and discretion have direct organizational impact (Hambrick 2007).

## 3 Discussion

The aims of this study were to examine two research questions concerning the microfoundations of organizational risk and the connections between macro‐ and microlevel concepts. With this study, I have drawn on the microfoundations perspective (Coleman 1990; Felin et al. 2015) to explain organizational risk. I populated the framework with conceptualizations of organizational contexts (culture), individuals (perceptions, personality, propensity, and behavior), and organizational risk (possibilities for negative outcomes at the level of the organization that disrupt their viability; Bromiley et al. 2001). Organizational culture creates micro contexts that influence the expression of individual‐level perception, personality, and propensity. These individual differences are enacted in risk‐taking behavior that aggregates and impacts on organizational risk by shifting possibilities for realizing opportunities or harm. The implication for theorizing is that models of organizational risk are enhanced by taking a cross‐level perspective that conceptualizes organizations as contexts for individual actions with organizational consequences.

**FIGURE 2:**
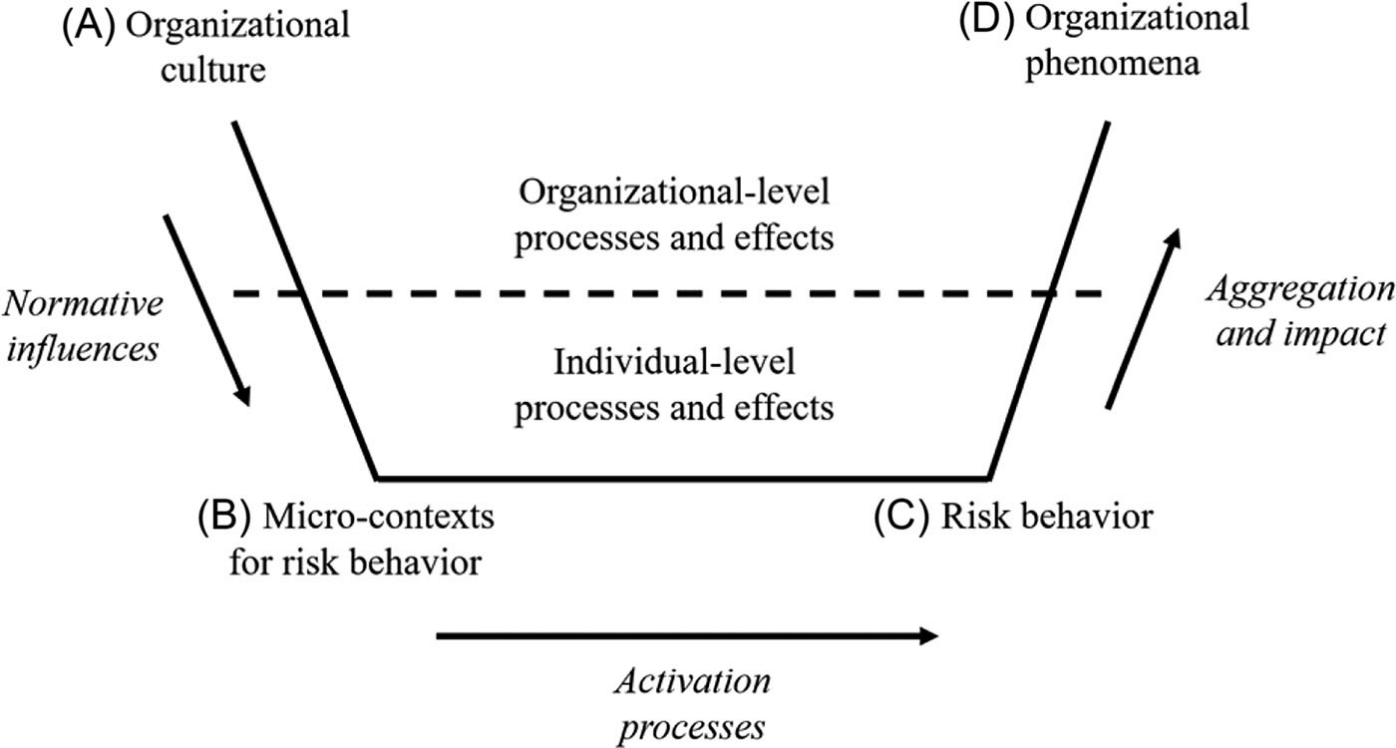
A microfoundations model of organizational risk.

Figure 2 depicts the model arising from my findings. Arrow 1 represents the macro–micro situational mechanisms arising from cultural norms that connect organizational culture with individual‐level contexts. Arrow 2 represents the micro–micro action formation mechanisms arising from the activation of individual‐level characteristics and their expression in behavior. Arrow 3 represents the transformational mechanisms that explain micro–macro connections in terms of aggregation and impact.

### 3.1 Theoretical Implications

The microfoundations perspective involves theorizing about the connections between different levels of analysis to explain organizational phenomena (Cowen et al. 2022). The insights arising from my study yield several theoretical implications for modeling organizational risk. The first implication concerns building theory to explain the situational macro–micro connections between organizational cultures and individuals. A core function of organizational culture is to foster internal integration that forms bonds between individuals (Schein 2017). Norms are key to such integration and to explanations of how organizational culture creates contexts for individual risk‐taking. Clear, consequential, and structured norms create strong situations that have powerful effects on behavior (Judge and Zapata 2015) and impose sanctions on people whose behavior does not meet those expectations (Cooke and Rousseau 1988). Such norms are oriented around core risk‐related values, such as ethics (Kaptein 2009) and safety (Hald et al. 2021; Le Coze 2019a).

The effects of norms can also be understood in relation to situational strength. Strong situations direct behavior by providing consistent clarity on what needs to be done, cues that motivate specific behaviors, and information about the consequences of adherence to or deviation from objectives (Meyer and Dalal 2009; Mischel 1977). A consequence is that the expression of individual differences is reduced, and behavior is homogeneous. Alternatively, weak situations, characterized by ambiguity and decentralized norms, permit the expression of individual differences (Mischel 1977). Such situations create behavioral variability that may be consistent within units and variable across units, or variable within and across units (Ostroff and Fulmer 2014).

The implication for theorizing concerns the capacity of models to explain the effects of organizational contexts on individuals. A focus on homogeneity begins with attention to what and how norms are developed and sustained (Schein 2017) and the organizational, social, and task cues managers create to activate individual characteristics and direct behavior toward valued outcomes (Tett et al. 2021). Doing so increases the predictability of organizational risks arising from organizational actors’ risk‐taking. The patterned profile of risk‐taking arising from variability in risk‐taking has a similar effect on organizational risk when profiles are aligned with objectives. Yet variability within and across units increases the difficulty of predicting organizational risk unless accompanied by a deep understanding of the concepts that form its microfoundations and the cross‐level processes that connect them.

Furthermore, models deepen explanations of organizational risk by considering it in relation to organizational objectives. At the individual level, risk‐taking may be a directional activity intended to achieve goals, such as wealth or excitement (Lauriola and Weller 2018). Risk‐taking is also intended to achieve a range of organizational outcomes including growth (Hoskisson et al. 2017), performance (Hardy et al. 2020), reputation (Bryce et al. 2024), and ethics (Bouchard and Dion‐Labrie 2024). Conversely, actors making organizational‐level decisions may avoid risks that are potentially detrimental, as seen in the public sector (Chen and Bozeman 2012). A microfoundations perspective on organizational risk is agnostic about whether risk‐taking should be high or low. More salient to organizational risk is the role situations play in shaping the expression of individual preferences in behavior (Meyer and Dalal 2009; Mischel 1977; Tett and Burnett 2003). The implication is that modeling organizational risk benefits from theorizing that connects cultural norms and practices with objectives. Such theorizing is extended by understanding the variability of objectives within and across units to explain the relationships between contexts and the individual‐level elements of a microfoundations model.

The second implication for theorizing concerns the action formation mechanisms that function at the microlevel. Micro contexts are created by individual‐level interactions with organizational characteristics (Bamberger 2008). These micro contexts may be similar across individuals, for example, when cultures are characterized by powerful norms and situations are strong (Mischel 1977). Alternatively, micro contexts may vary at the individual level due to weak situations (Meyer and Dalal 2009). Whether situations are strong or weak, managers may align behaviors with specific objectives through organizational, social, and task cues (Tett and Burnett 2003). Doing so enables managers to recognize and reward the expressions of traits and other individual differences that contribute to achieving organizational goals (Tett and Fisher 2021; Tett et al. 2021). The implication for models of organizational risk is that incorporating the individual‐level interplay between organizational contexts and responses to them increases their accuracy.

Indeed, when cues are specific, trait activation theory explains individual‐level processes more accurately than situational strength theory (Judge and Zapata 2015). Such theorizing is enriched by recognizing the role of managers in shaping action formation mechanisms. By altering cues and the values attached to specific behaviors, managers create possibilities for directing risk‐taking (Tett and Guterman 2000). For example, variable incentive schemes encourage the expression of stronger risk‐taking preferences than fixed pay schemes. Managers who change incentive schemes (i.e., organizational cues) also change the likelihood of risk preferences being enacted (Fulmer and Shaw 2018). Thus, managers create conditions for individuals to express themselves in risk‐taking behavior. The sensitivity of activation processes and their responsiveness to short time frames (Woods et al. 2013) create frequent possibilities for managers to use cues that shift the direction and extent of risk‐taking in ways that shape the connections between microlevel contexts and actions.

The third implication for theorizing concerns the transformational mechanisms that connect individual risk‐taking and organizational risk. My study has identified three ways these transformational mechanisms occur: (1) the aggregation of homogeneous actions, which may range from low risk‐taking to high risk‐taking; (2) the variability created by heterogeneous actions across organizations that also have aggregate effects; and (3) the organizational‐level impact of risk‐taking by individuals who have high levels of discretion and autonomy. Making such links is typically the most challenging element of any microfoundations model (Felin et al. 2015). Hence, identifying these connections expands theorizing about organizational risk by elucidating three mechanisms linking the micro‐ and macrolevel concepts.

Examining how variability functions adds depth to explanations of these connections. The inclusion of objectives in a model of organizational risk is complemented by the inclusion of systems that enable coordination and provide oversight. Yet the effectiveness of such systems is often hampered by structural impediments (Bantleon et al. 2021). In addition, senior leaders influence their organizations directly through their enacted risk preferences (Li and Tang 2010). Authority and discretion are key to understanding such influence processes (Neely et al. 2020). Considering governance structures, such as board composition and functioning (Renn et al. 2022), contributes to explanations of the extent to which organizational risk reflects leaders’ risk‐taking, as well as the scope for autonomous actors lower in the hierarchy to impact on their organizations. Furthermore, knowledge integration processes, such as developing knowledge networks, establishing common ground, and areas of specialization (Barley et al. 2018), provide the oversight necessary to comprehend and change individual risk‐taking, its antecedents, and consequences. Thus, modeling organizational risk benefits from considerations of how it is linked with organizational processes that create homogeneity or variability.

Furthermore, my examination of organizational risk has implications for the broader field of risk science, a distinct line of enquiry that informs risk assessment, communication, management, and governance (Aven and Zio 2021). Risk science emphasizes the importance of interdisciplinary approaches to understand risks (Aven 2024b). Risk science also acknowledges how complex risks require interpretation and generate subjective views to guide actions (Aven 2024a). These developments deepen insights into risks by going beyond a positivistic perspective on risk and the associated focus on quantitative or technical analysis by embracing uncertainty, as seen in aviation (Garrick and Mosleh 2020). With this study, I show how theories from psychology populate a microfoundations framework and enhance explanations of organizational risk. In doing so, I contribute to risk science by illuminating processes that occur within organizations. The implications are significant given that organizations and organizing processes are at the heart of risk assessment, communication, management, and governance.

### 3.2 Future Research

Several implications for future research arise from the proposed microfoundations model. A first possibility concerns empirical examination of the model. I have suggested organizational risk is a consequence of organizational culture, individual‐level interpretations of cultural norms that create contexts for the expression of personality traits and other relevant individual differences, and the effects of risk‐taking across an organization. Future research could examine this proposed sequence by assessing each of these concepts as well as the connections between them. For example, examining the microfoundations of cybersecurity involves assessing individual cyber‐risk assessments in relation to several types of data breach, personal online behavior, and support for specific cybersecurity policies (Kostyuk and Wayne 2021). Similar methods could be applied to the proposed model of organizational risk and would enable empirical testing of the concepts, as well as examinations of the macro–micro connections and their possible interactions.

A second opportunity for future research concerns the possibility of changing organizational risk by focusing on the processes that create cross‐level effects between the core concepts. Although prior studies examine the effects of context on individual behavior (Tett and Fisher 2021; Tett et al. 2013), the links between individual risk‐taking and organizational risk are less well understood. Such research is important because studies of the connections between lower‐ and higher‐level effects benefit from developing theorizing that is integrated across levels (Cowen et al. 2022). My review has identified three ways individual risk‐taking influences organizational risk, namely, two forms of cross‐level aggregation and one form of impact via discretion and autonomy. The nature of these connections also provides insights into how organizational risk can be altered. Attention to what and how norms are fostered sheds light on the pressures to behave in particular ways (Hald et al. 2021; Reader et al. 2015). Further research into these processes would extend knowledge about the specific connections between organizational culture and individual beliefs about the value of risk‐taking and its consequences for organizational risk.

Such research could be extended by focusing on the role of leaders in shaping the microfoundations of organizational risk. All organizations are exposed to risk and sit somewhere on a spectrum ranging from low risk to high risk. Positioning organizations on that spectrum is the role of leaders who have responsibilities for influencing culture and the capacity to do so (Kim et al. 2022). Founders establish organizations that reflect their values and motives by developing central assumptions and creating systems that foster their acceptance, setting up reward systems, and influencing the selection and retention of people in positions of authority (Schein 2017). Leaders’ personality traits influence the cultures they create (O'Reilly et al. 2014). The corollary is that cultures are not independent of agency (Kim et al. 2022). Rather, leaders shape cultures and create possibilities for creating cultures where risk orientation supports the achievement of organizational objectives. Theorizing that makes connections between contexts and the leaders who shape them provides insights into how cultures are formed and changed, as well furthering understanding of how individual‐level characteristics and behaviors influence organizational‐level outcomes.

A third research pathway involves expanding the microfoundations modeling by broadening the range of concepts included. Although I have selected concepts that are typical representations of their level of analysis in the psychology literature (i.e., organizational culture as a macrolevel concept, and personality, perceptions, propensity, and behavior as microlevel concepts), other possibilities from the same and alternative literatures exist. Examining the effects of strategy on norms and individual‐level behaviors would enhance explanations of organizational risk. Further studies could also explore different aspects of individual‐level decision processes and behavior. For example, advances in risk science highlight the role of sensemaking in interpreting risks and shaping individual as well as collective actions (Taarup‐Esbensen 2019, 2023). Convergence also exists on how the notions of interpretation and variability inform understanding of risk in the science and technology studies literature (Downer 2017). Research that takes an expanded view by drawing on additional frameworks and concepts will further theoretical development and refine organizational risk models.

## 4 Conclusion

By drawing on the microfoundations perspective (Felin et al. 2015), I generate three insights into organizational risk. First, my study shows how macro‐ and microlevel theorizing extends theorizing about how organizational risk emerges from internal norms, practices, processes, and behaviors. Second, I elucidate the mechanisms that explain the connections between macro–micro, micro–micro, and micro–macro phenomena. In doing so, I identify several ways that individual risk‐taking influences organizational risk, namely, the aggregation of homogenous and variable risk‐taking within and across units as well as the organizational‐level impact of risk‐taking by people with autonomy and discretion. Third, I contribute to the risk science literature by showing how theories from psychology contribute to the interdisciplinary theorizing required to understand the complexities of organizational risk.

## Conflicts of Interest

The author declares no conflicts of interest.

## Data Availability

Data sharing is not applicable to this article as no datasets were generated or analyzed during the current study.
